# From abstract painting to action painting: rethinking embodied simulation in aesthetic experience

**DOI:** 10.3389/fnhum.2025.1643718

**Published:** 2025-08-20

**Authors:** Joung A. Eom, Junhee Kim

**Affiliations:** Department of Aesthetics, Seoul National University, Seoul, Republic of Korea

**Keywords:** embodied simulation, mirror neuron system (MNS), abstract painting, aesthetic experience, neuroaesthetics

## Introduction

Neuroaesthetics is a field of study that investigates the neural mechanisms underlying aesthetic experience (Pearce et al., 2016). The concept of “aesthetic experience” has long been a subject of philosophical debate. Advances in neuroimaging now permit researchers to explore brain regions involved in the aesthetic experience of art (Petcu, 2018). Nevertheless, both neuroaesthetics and philosophy continue to grapple with the persistent challenge of clearly articulating what constitutes an aesthetic experience, a difficulty that underscores the complexity of this phenomenon (Nadal et al., 2012; Carroll, 2012). In this paper, we use “aesthetic experience” to refer broadly to any perceptual response to artworks, understood as a distinct class of experience elicited by objects intentionally set apart from ordinary life.

Freedberg and Gallese (2007) introduced an embodied simulation within the context of art perception, proposing that the aesthetic experience of observing depicted actions and the traces of an artist's physical gestures involves the activation of the human mirror neuron system (MNS). Mirror neurons—first identified in macaques—are a class of visuomotor neurons that discharge both when an individual executes an action and when they observe another individual performing the same action (Rizzolatti and Craighero, 2004). Neuroscientist Gallese (2005) argued that the activation of the MNS enables observers to internally replicate the perceived action within their neural circuitry, forming an action-observation network that functionally links perception and motor processes. This mechanism, which Gallese termed embodied simulation, involves the internal reenactment of observed actions within the observer's motor system—*embodied* in the sense that the simulation is grounded in the observer's motor capacities (Gallese and Sinigaglia, 2011).

Sbriscia-Fioretti et al. (2013) present intriguing findings on the significance of embodied simulation of brushstrokes, traces of the artist's movement, in abstract paintings. Influenced by art critic Greenberg (2018)'s Modernism theory, the aesthetic experience of abstract paintings has traditionally been considered within the visual domain. Nevertheless, the study by Sbriscia-Fioretti et al. demonstrates that the beholders not only recognize the creative movement of the artist but also mentally draw upon the painting themselves while viewing. It reveals the inclusion of bodily experience in viewing abstract paintings, thereby contributing significantly to the broaden understanding of aesthetic experience for abstract paintings.

However, Sbriscia-Fioretti et al. presented abstract paintings as traces of hand movement. Abstract paintings are produced by hand movement, yet the perception of such traces is not the same as the perception of abstract paintings, as they are not simply a collection of brushstrokes. Therefore, the results cannot automatically be assumed as a reaction to abstract paintings. Whether the result can be applied to abstract paintings and could be considered a distinctive aspect of responses to such paintings needs to be examined. We discuss these issues and hypothesize that the embodied simulation of brushstrokes is a discrete aesthetic experience for action paintings, a subset of abstract paintings, rather than abstract paintings in general.

## Exploring the relevance of embodied simulation to artworks

In their study, Sbriscia-Fioretti et al. anticipated that the participants could perceive a certain degree of dynamicity from brushstrokes, the traces of hand movement, in Franz Kline's abstract paintings *Suspended* 1953, *Painting Number 2* 1954, *Painting Number 7* 1952. The analysis of the event-related potential (ERP) sources showed motor activation. The authors interpreted these findings with Gallese's embodied simulation theory, proposing that the participants' brains were simulating the hand movements implied by the brushstrokes even though they remained physically still. They argued that this simulation was induced specifically by the artist's ability to emphasize brushstrokes, suggesting that it is a distinctive aspect of the response to abstract paintings (Sbriscia-Fioretti et al., 2013).

However, Heimann et al. (2013) showed that embodied simulation can also be triggered by observing any traces of hand movements, such as “scribbles.” It happened without an artist's ability to emphasize brushstrokes; therefore, it is hard to believe that embodied simulation of brushstrokes is a distinctive experience for such paintings. Moreover, responses to observed actions depend on more than dynamic cues alone. Iacoboni et al. discovered that observing actions within a context resulted in larger motor responses than observing simple actions. The context offered clues to recognize the intention of the action, resulting in stronger reactions. Although limited to immediate, stimulus-linked intentions, the study showed that embodied simulation plays a role in the low-level processing of observed actions (Iacoboni et al., 2005). Considering the enhanced motor response to action with intention rather than to simple action, it is possible that traces of hand movement were not the only cause for embodied simulation in viewing Kline's paintings.

Sbriscia-Fioretti et al. (2013) presented Kline's paintings as brushstrokes. Nonetheless, Kline's paintings were not perceived as brushstrokes but as artworks. In a questionnaire, the authors asked participants if the images seen in the experiment were authentic artworks. The results showed that participants recognized them as real artworks (Sbriscia-Fioretti et al., 2013). It indicates that Kline's paintings were viewed as artworks. It is possible that knowing them as artworks functioned like the context and provided clues about the intention of the perceived action.

If we will consider the perceived action in scribbles and abstract paintings, we can substitute observing scribbles for observing simple hand movements and observing abstract paintings for those presented in a context. Therefore, we can expect a greater motor reaction when viewing abstract paintings than when observing scribbles. Both Kline's paintings and scribbles elicited motor responses, but for different reasons. The action that stimulated the motor areas in Sbriscia-Fioretti et al.'s experiment was not a simple hand movement, but a movement that was intended to create artwork. The embodied simulation that occurs while viewing Kline's paintings is not solely in response to the traces of hand movement, but also to Kline's intention to create artwork.

In Kline's paintings, brushstrokes and artistic nature are inseparable. Although Sbriscia-Fioretti et al. were unaware, the control stimuli used in the study not only lacked the nature of being traced but also lacked the status of being artworks. It means that the response elicited by Kline's paintings inherently encompasses their applicability as artworks. Our discussion clarified the underlying cause for increased motor activity in response to abstract paintings.

## Examining the distinctiveness of embodied simulation in aesthetic experience

We discussed the relevance of embodied simulation to brushstrokes in Kline's paintings as a reaction to artworks. However, it is insufficient to claim that such simulation is a distinctive reaction to abstract paintings. Expanding on the investigation by Sbriscia-Fioretti et al., Eom and Lee examined whether embodied simulation is also triggered by Se Ok Suh's abstract oriental paintings—*Person* 1990, *Mother and Son* 2000, *and People* 1997—which prominently feature thick black lines reminiscent of Kline's style. The analysis of the EEG data revealed the absence of mu rhythm suppression, indicating the absence of embodied simulation (Eom and Lee, 2018). Although mu suppression is widely used as an index of the mirror neuron system (MNS) activity (Perry and Bentin, 2009; Fox et al., 2016), its validity has been questioned due to the spectral overlap with alpha rhythms, which are linked to general cognitive and attentional processes (Hobson and Bishop, 2016). Eom and Lee (2018) assessed both mu and alpha rhythms, thereby demonstrating that alpha activity was not a confounding factor in their findings. Thus, their findings indicated that embodied simulation was absent (Eom and Lee, 2018).

Despite both Kline's and Suh's paintings featuring clear brushstrokes, only one set elicited embodied simulation, as summarized in Table 1. Suh's study focused on portraying the human figure in an abstract manner. These paintings are considered successful examples of presenting both human forms and distinct brushstrokes (Kim, 2015). By definition, abstract paintings do not depict subjects in a strictly realistic or illusionistic manner. Nevertheless, the degree of abstraction can vary, allowing the subject matter to remain at least partially recognizable in some cases. As such, abstract painting spans a broad continuum—from studies that are abstract yet still representational, such as Willem de Kooning's *Woman I* 1950–1952, to entirely non-representative compositions, such as Jackson Pollock's *Autumn Rhythm* 1950.

**Table 1:** Comparison of experimental designs and key findings on embodied simulation in abstract and action paintings.

**Features**	**(Sbriscia-Fioretti et al. 2013)**	**(Eom and Lee 2018)**
Stimuli used in the study	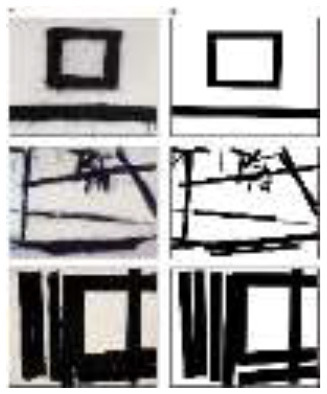 Reprinted from Sbriscia-Fioretti et al. (2013), under the terms of the Creative Commons Attribution License (CC BY). © Franz Kline / Artists Rights Society (ARS), New York - SACK, Seoul, 2025 Stimuli used: (Left) Original paintings by Franz Kline (Top to bottom: *Suspended*, 1953; *Painting Number 2*, 1954; *Painting Number 7*, 1952) (Right) Modified versions with dynamic components removed	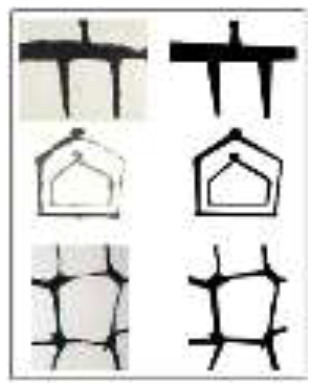 Reprinted with permission from Eom and Lee (2018) Stimuli used: (Left) Original paintings by Se Ok Suh's (Top to bottom: *Person*, 1990; *Mother and Son*, 2000; *People*, 1997) (Right) Modified versions with dynamic components removed.
Visual elements	**Mark-making (brushstrokes)**	**Mark-making (brushstrokes) and abstract human figures**
Brain activity measurement	Event Related Potentials (ERPs). Source localization was conducted using standardized Low-Resolution Brain Electromagnetic Tomography (s-LORETA) to identify cortical areas.	EEG mu (8-14 Hz) and alpha (8–14 Hz) rhythms.
Behavioral tests	Ratings on aesthetic appraisal, perceived movement, familiarity, and artistic nature.	Ratings on aesthetic appraisal, perceived movement, familiarity, and artistic nature.
Key findings (EEG)	▸**Greater fronto-central ERP amplitudes observed for original paintings vs. modified stimuli (~300 ms post-stimulus)**. ▸ Participants were generally unfamiliar with both stimulus types.	▸**No significant mu or alpha suppression differences between original and modified stimuli**. ▸ Participants were divided into two groups based on the average familiarity scores for the three paintings. However, group familiarity levels did not significantly interact with neural response.
Key findings (Behavioral)	▸ Behavioral ratings (aesthetic appraisal and perceived movement) were significantly higher for paintings compared to modified stimuli. ▸ Participants consistently perceived the paintings as original artworks, whereas the modified stimuli were not regarded as such.	▸ Behavioral ratings (aesthetic appraisal and perceived movement) were significantly higher for paintings compared to modified stimuli. ▸ Participants consistently perceived the paintings as original artworks, whereas the modified stimuli were not regarded as such.

This table compares the experimental stimuli, visual characteristics, methods, and results of the studies conducted by Sbriscia-Fioretti et al. and Eom and Lee, highlighting neural responses related to embodied simulation.

The argument of Sbriscia-Fioretti et al. was based on limited paintings by a single artist, which inherently restricted the generalizability of their claims regarding abstract painting as a whole. Art historian Gombrich (1969) noted the alternative relationship between brushstrokes and represented content within a painting. Accordingly, brushstrokes are not noticeable, while represented content, such as a human figure, is recognized.

It is important to recognize that the brushstrokes in Franz Kline's paintings are more than mere visual elements; they are integral to the performative nature of his study. Kline's paintings are representative of what art critic Harold Rosenberg termed “Action Painting.” According to Rosenberg, in action painting, “the painter no longer approached his easel with an image in his mind; he went up to it with material in his hand to do something to that other piece of material in front of him” (Rosenberg, 1952). This approach signaled a shift in emphasis from the depiction of a subject to the enactment of painting itself as a meaningful event. The canvas was no longer a space for representation but an “arena in which to act” (Rosenberg, 1952). Within this framework, mark-making—whether through brushstrokes, drips, or splatters—served as the visible trace of the artist's physical engagement with the medium. This mark-making was not intended to represent external imagery, but to record the act of creation, rendering the painting process itself visible. As such, mark-making in action in painting transcended formal aesthetics and became a direct manifestation of the artist's performative gesture (Rosenberg, 1952).

Sbriscia-Fioretti et al. claimed that an artist's ability to emphasize brushstrokes that evoke embodied simulation is what distinguishes abstract paintings. While this may not apply universally to all abstract art, it is particularly relevant to action painting. Throughout art history, it is relatively rare—even within abstraction—for paintings to foreground brushstrokes to the extent that they become the primary object of perception. However, in action painting, the implication of the artist's physical movement through mark-making is fundamental. The mark-making itself becomes the central content, which is what sets action painting apart.

As Sbriscia-Fioretti et al. argued, the artist must have emphasized mark-making to make the viewer focus on them. The presence of embodied simulation in viewers of Kline's study suggests that this effort was successful—it implies that the viewer is not merely seeing the result, but mentally simulating the artist's action. Embodied simulation, in this context, becomes an aesthetic experience deliberately induced by the artist. Therefore, we propose that embodied simulation is not a general response to abstract art, but a unique aesthetic feature of action painting.

Moreover, the embodied simulation might lead the viewers to aesthetic pleasure. In action paintings, there is nothing but mark-making. Clement Greenberg highly valued this visual configuration, considering it to encapsulate what is essential in painting (Greenberg, 1982). However, this perspective does not explain the motivation behind the beholding of action paintings. Leder et al. (2012) and Ticini et al. (2014) demonstrated that aesthetic preference is linked to motor execution, proposing embodied simulation as a source of aesthetic pleasure alongside perceptual and reward-related systems. Finisguerra et al. further explored the relationship between embodied simulation and dispositional empathy, offering an explanation for how even naïve viewers may experience aesthetic pleasure. These findings suggest that embodied simulation may serve as a potential source of aesthetic pleasure when viewing action painting.

## Conclusion

In this article, we proposed that embodied simulation of mark-making is a significant aesthetic experience for action paintings. Additionally, there are studies suggesting that bodily experience may influence aesthetic appreciation. Converging all these together contributes to enhancing the understanding of the aesthetic experience and aesthetic pleasure derived from action paintings. While EEG/ERP measures offer only indirect evidence and may be limited in capturing complex aesthetic or emotional responses, our argument is based on a limited number of empirical studies. This study nonetheless underscores the importance of further investigation into aesthetic experience across diverse forms of art.
